# Effect and Mechanism of *Bifidobacterium animalis* B94 in the Prevention and Treatment of Liver Injury in Rats

**DOI:** 10.3389/fcimb.2022.914684

**Published:** 2022-06-29

**Authors:** Tianfang Zhang, Jie Wang, Zhao Yao, Lingmei Ni, Yifan Zhao, Shuang Wei, Zuobing Chen

**Affiliations:** ^1^Department of Rehabilitation Medicine, The First Affiliated Hospital, School of Medicine, Zhejiang University, Hangzhou, China; ^2^The Second Affiliated Hospital and Yuying Children’s Hospital, Wenzhou Medical University, Wenzhou, China; ^3^Infection Prevention and Control Department, The First Affiliated Hospital, School of Medicine, Zhejiang University, Hangzhou, China

**Keywords:** liver injury, gut microbiota, metabolome, *Bifidobacterium animalis* B94, probiotics

## Abstract

**Objective:**

To investigate the effect of *Bifidobacterium animalis* B94 on the prevention and treatment of liver injury in rats and to elucidate the underlying mechanism of this relationship.

**Methods:**

Specific pathogen-free (SPF) rats were selected as the healthy control group, liver injury group and B94 treatment group, with 6 rats in each group. After the model was established, the experimental animals were tested for serum liver function indicators, gut microbiota composition, metabolite composition, and histopathology.

**Results:**

The albumin/globulin ratio and serum TBA, alanine aminotransferase, aspartate aminotransferase, and indirect bilirubin levels in the B94 treatment group were significantly lower than those in the liver injury group. 16S rRNA analysis showed that the gut microbiota of the three groups of rats were significantly different. Metabolic profile analysis showed that there were significant differences in the gut metabolomes of the three groups. Haematoxylin–eosin staining of the intestinal mucosa and liver tissues showed that the degree of liver and intestinal tissue damage in the B94 treatment group was significantly lower than that in the liver injury group.

**Conclusion:**

*Bifidobacterium animalis* B94 can affect the process of liver injury in rats by improving liver function, reducing intestinal damage, and regulating gut microbiota and metabolite production.

## Introduction

The liver has the functions of synthesis, detoxification, metabolism, secretion, biotransformation, and immune defence and is one of the important organs of the human body. When hepatocytes are severely damaged by viruses, alcohol, drugs, etc., they become necrotic in large numbers, resulting in serious impairment or failure of the abovementioned functions. Then, liver failure occurs and manifests as a group of clinical syndromes, including impaired coagulation and jaundice, hepatic encephalopathy, ascites, etc. Liver failure is a common clinical syndrome of severe liver disease, with rapid progression, a high mortality rate and a poor overall prognosis.

The intestine is the largest bacterial reservoir in the body, and it decomposes, utilizes, transforms and produces a large number of exogenous substances, participates in the enterohepatic circulation of bile acids and other substances, promotes tissue development, regulates immunity, and prevents invasion and infection by foreign microorganisms through nutritional competition and occupancy protection (Guarner and Malagelada, 2003; Backhed et al., 2005). Once microbiota dysbiosis occurs, the intestinal mucosal barrier can become disrupted, which will cause immune dysregulation of the host, and some pathogens can even translocate to other organs, such as the liver and induce disease (Yan et al., 2011; Dapito et al., 2012; Lv et al., 2014). Liver injury can cause intestinal motility, immune and metabolic changes and intestinal mucosal barrier damage, which can in turn lead to structural changes in the gut microbiota, endotoxaemia, and bacterial translocation (Acharya and Bajaj, 2017). Therefore, ameliorating gut microbiota dysbiosis has become an important element in the prevention and treatment of liver injury or liver failure. However, there is a general lack of corresponding products worldwide.

Studies have shown that *Bifidobacterium animalis* B94 can alleviate *Helicobacter pylori*-associated gastritis through the production of organic acids, autolysins, mucins and bacteriocins and immunomodulatory effects and that it promotes a high eradication rate for *Helicobacter pylori*-induced diarrhoea (Zhang et al., 2008; Donkor et al., 2012; Cekin et al., 2017). In regulating the immune system, *B. animalis* B94 can induce substantial secretion of pro- and anti-inflammatory cytokines from normal peripheral blood monocyte-derived monocytes and macrophages and effectively induce Th17 and Treg cell differentiation for immunomodulation (Donkor et al., 2012). B94 can also improve intestinal microecological dysregulation, inhibit the adhesion and colonization of intestinal pathogenic bacteria through multiple pathways, and be used for the treatment of irritable bowel syndrome (Basturk et al., 2016).

In this study, we investigated the effect of *B. animalis* B94 in the prevention and treatment of liver injury and the potential mechanism of action using a rat model of liver failure. The results of this study are important for research on the molecular mechanism and clinical application of B94.

## Materials and Methods

### Microorganism Preparation

The *Bifidobacterium animalis* B94 strain was obtained from Lallemand (Ontario, Canada). B94 was inoculated into MRS liquid medium (Thermo Fisher, Shanghai, China) anaerobically at 37°C for 24 h to logarithmic growth phase. Bacteria were collected by centrifugation at 5000 ×g for 10 min, washed three times and resuspended in sterile saline (0.9% (w/v)) at a concentration of 3×10^9^ CFU/mL.

### Animal Experimental Design

Eighteen specific pathogen-free (SPF)-grade Sprague Dawley (SD) male rats (Shanghai SLAC Laboratory Animal Co., Ltd., China), weighing between 200-300 g, were housed at room temperature (22 ± 2°C) and given ad libitum access to food and water, with 12/12 h alternating between day and night (light and dark) each day. After a 2-week period of cohousing, the rats were randomly divided into three groups of six animals each, consisting of the healthy control group (HC): gavage with saline + intraperitoneal injection of saline; liver injury group (GalN): gavage with saline + intraperitoneal injection of D-galactosamine; and B94 treatment group (B94+GalN): gavage with B94 + intraperitoneal injection of D-galactosamine. All the experimental procedures followed the National Institutes of Health (NIH) Guide for the Care and Use of Laboratory Animals. The study protocol was approved by the Animal Experimentation Ethics Committee of Zhejiang University.

For the first seven days, rats were gavaged with saline or 1 mL of a freshly prepared B94 solution (3 × 10^9^ CFU/mL) once per day, according to the grouping described above. On day 7, the rat liver injury model was constructed by intraperitoneal injection of saline or 1.1 g/kg D-galactosamine (Sigma, Saint Louis, MO, USA), according to the grouping. At 24 h after D-galactosamine injection, the rats were anaesthetized with 400 mg/kg chloral hydrate (Sigma, Saint Louis, MO, USA) delivered intraperitoneally, and the rats were operated on with strict aseptic technique. The liver and colon tissues were fixed in 10% paraformaldehyde and embedded in paraffin for pathological specimen preparation. Finally, stool was taken from the rectum for 16S rRNA and metabolome assays.

### Liver Function Assay

The serum was separated by centrifugation at 3000 rpm for 10 min at room temperature, and 400 µL of serum was collected and analysed by a fully automated biochemical analyser (Hitachi 7600-210; Tokyo, Japan) to determine alanine aminotransferase (ALT), aspartate aminotransferase (AST), alkaline phosphatase (ALP), total protein, globulin, albumin, total bile acid (TBA), total bilirubin (TBil), direct bilirubin (DBil), indirect bilirubin (IBil), γ-glutamyl transferase (GGT) and glycylproline dipeptidyl aminopeptidase (GPDA) levels.

### Serum Cytokine Assay

We used a Bio-plex Pro™ rat cytokine 23-plex assay (Bio-Rad, Hercules, California, USA) kit to determine serum IL-1α, IL-1β, IL-2, IL-4, IL-5, IL-6, IL-7, IL-10, IL-12, IL-13, IL 17, IL-18, colony-stimulating factor (G-CSF), granulocyte-macrophage colony-stimulating factor (GM-CSF), growth-regulating α protein (GRO/KC), interferon-γ (IFN-γ), macrophage colony-stimulating factor (M-CSF), monocyte chemoattractant protein 1 (MCP-1), macrophage inflammatory protein 1α (MIP-1α) MIP-3α, tumour necrosis factor-α (TNF-α), vascular endothelial growth factor (VEGF), and regulated on activation normal T-cell expressed and secreted (RANTES) levels.

### 16S rRNA Sequencing and Analysis

DNA was extracted from 0.2 g of stool using a QIAamp Fast DNA Stool Mini Kit (Qiagen, Valencia, USA). GGACTACHVGGGTWTCTAAT-3’) for PCR amplification of the 16S rRNA V3+V4 region (Lv et al., 2021a). The PCR products were purified using AMPure XPbeeds (Agencourt, Beckman Coulter, USA), and the library was quantified by real-time quantitative PCR. Paired-end sequencing (2 × 300 bp) was performed using the Illumina MiSeq platform (Illumina, San Diego, CA). Raw reads were cleaned, filtered and then merged using FLASH (v1.2.11). Vsearch (v2.3.4) was used to select operational taxonomic units (OTUs) with sequence similarity greater than 97%. OTU clustering, identification based on the RDP database and the NCBI-16S database, and subsequent statistical analysis of microbial diversity and differential enrichment were analysed using QIIME (v1.9.1).

### Detection and Analysis of Faecal Metabolites

Samples for metabolite assays were pretreated as described previously (Jiang et al., 2021; Lv et al., 2021b). Briefly, 20 mg of faeces was added to 800 μL of precooled chromatography grade methanol and then homogenized three times using a Precellys Evolution instrument (Bertin Technologies, USA) at 5,000 rpm for 30 s with 15 s intervals between the rounds for extraction. After centrifugation at 14,000 rpm for 15 min, the supernatant was filtered through a 0.22 µm membrane, and 20 µL of heptadecanoic acid (1 mg/mL, Sigma-Aldrich, St. Louis, MO, USA) was added to the filtrate as an internal reference and then dried under nitrogen at room temperature. After drying, the samples were methoxymated with methoxypyridine (Sigma-Aldrich, St. Louis, MO, USA) and trimethylsilylated with N,O-bis(trimethylsilyl)acetamide containing 1% trimethylsilyl chloride. The pretreated samples were analysed with an Agilent 7890A-5975C GC-MS system (Agilent, USA). The downstream data were compared with the NIST 17 database programmatically to identify the corresponding metabolites (matching score ≥ 80%).

### Histopathological Evaluation

The fixed and embedded liver and colon samples were sectioned (2 µm) and stained with haematoxylin-eosin (HE). Sequentially, the images were observed under a microscope to evaluate the liver damage according to the histopathological activity index (HAI) method (Knodell et al., 1981; Lv et al., 2021b) and the intestinal epithelium abnormalities according to the reference reported by C.J. Chiu et al. (1970).

### Statistical Methods

For the comparison of liver function, liver and colon histopathology scores, gut bacterial α diversity and faecal metabolites among groups, the Shapiro-Wilk test was first used to determine whether the data of each group conformed to a normal distribution. One-way ANOVA followed by the Student-Newman-Keuls method was used to compare any two data sets that were normally distributed; otherwise, the Mann-Whitney U test was used. The Wilcoxon rank sum test combined with the Benjamini-Hochberg method was used to compare the relative abundances of each taxonomic level of gut bacteria between groups. Correlations between variables were analysed by Spearman’s rank correlation test. *P* < 0.05 was considered statistically significant.

## Results

### *Bifidobacterium animalis* B94 Alleviated D-Galactosamine-Induced Abnormal Liver Function and Immune Dysfunction

**Figure 1:**
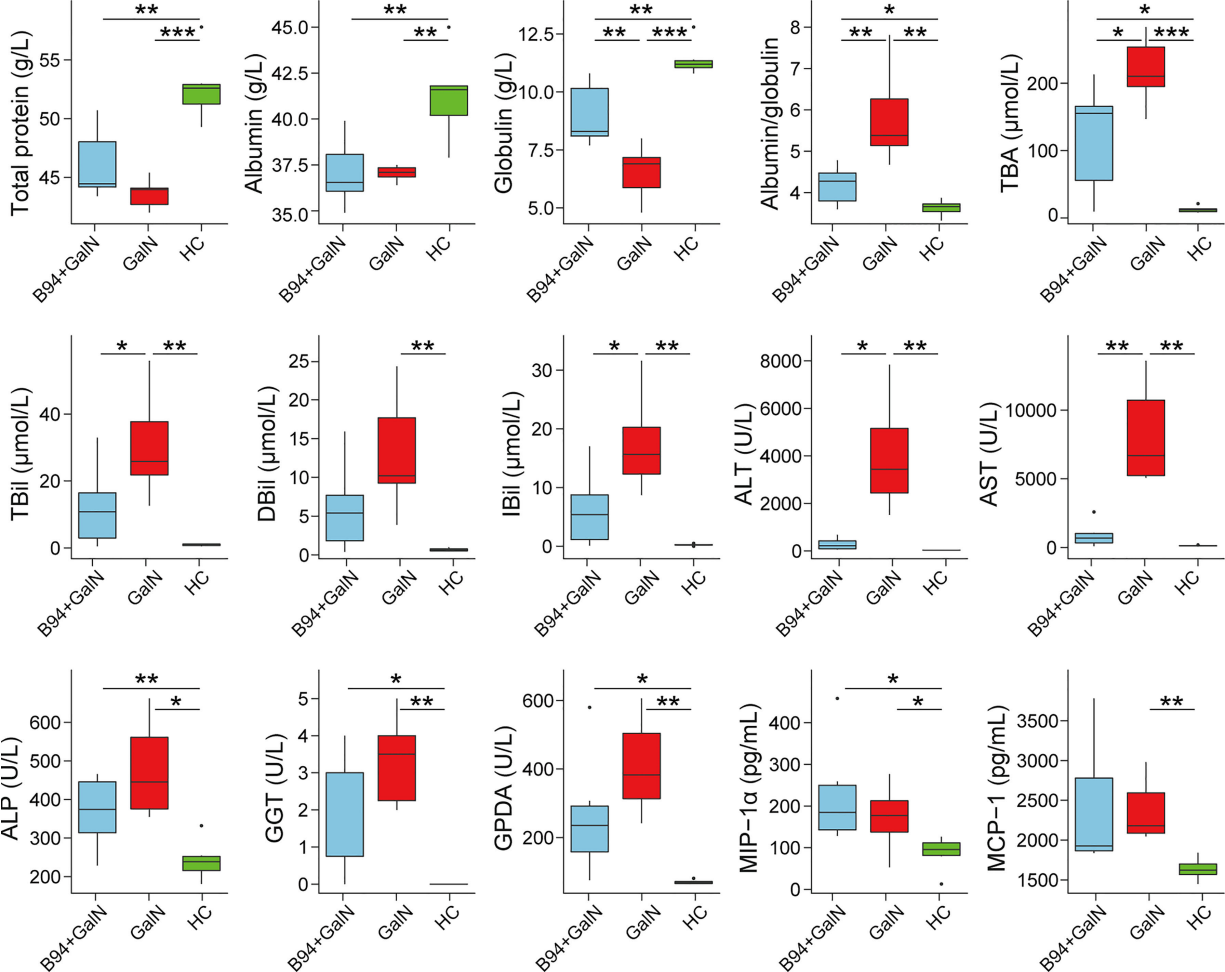
B94 treatment alleviates D-galactosamine-induced abnormal liver function indicator levels and immune dysfunction. * *P* < 0.05; ** *P* < 0.01; *** *P* < 0.001.

After D-galactosamine injection, the total protein, albumin and globulin levels were significantly lower in the GalN group rats than in the HC group rats, while the albumin/globulin ratio and ALT, AST, ALP, TBA, TBil, DBil, IBil, GGT and GPDA levels were significantly higher in the GalN group rats than in the HC group rats (**Figure 1**). B94 treatment significantly ameliorated the decrease in globulin levels induced by D-galactosamine, as well as the increase in the albumin/globulin ratio and ALT, AST, TBA, TBil and IBil levels.

To investigate the effect of B94 on immune function in rats, we examined the levels of 23 cytokines in rat serum. The results showed that D-galactosamine injection caused an upregulation of MCP-1 and MIP-1α levels in rat serum compared with that in healthy controls. B94 gavage attenuated the D-galactosamine-induced elevation of MCP-1 levels so that the difference with the HC group was no longer significant.

### *Bifidobacterium animalis* B94 Ameliorated Liver and Intestinal Pathological Damage Caused by D-Galactosamine

**Figure 2:**
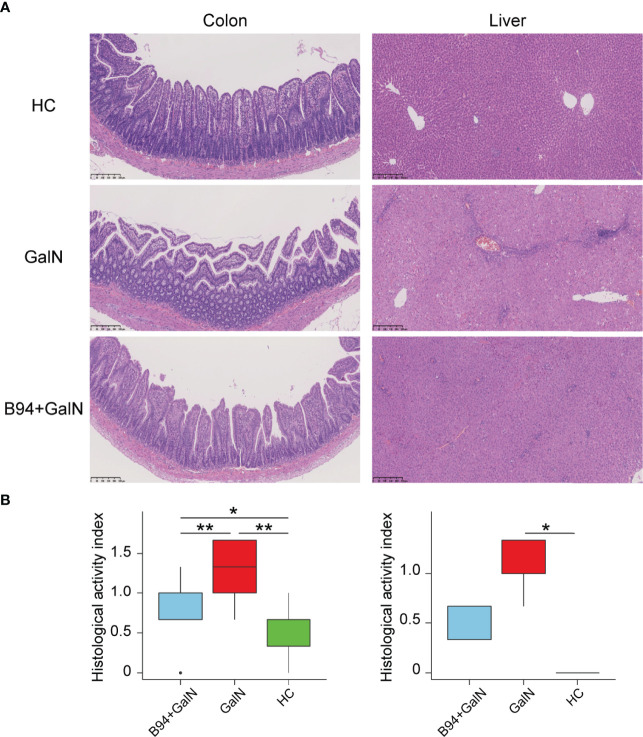
Representative images of colon and liver samples stained with HE **(A)** and histological scores **(B)** indicating that B94 treatment partially alleviated D-galactosamine-induced organ damage. * *P* < 0.05; ** *P* < 0.01.

The HE staining results of liver samples showed inflammatory cell infiltration and large areas of hepatocyte necrosis in liver sections of the GalN group rats, and liver injury was significantly reduced in liver sections of the B94+GalN group rats (**Figure 2A**). The HAI scores (**Figure 2B**) also showed that B94 treatment tended to alleviate the increase in the degree of liver damage caused by D-galactosamine (*P* = 0.057 in B94+GalN *vs.* GalN). The HE staining results of colon samples showed that the epithelium was damaged, with broken or missing villi and disintegrated crypts, in the GalN group (**Figure 2A**). In the B94+GalN group, this damage was ameliorated, and significantly lower organ damage scores were also observed compared with the GalN group (**Figure 2B**).

### *Bifidobacterium animalis* B94 Alleviates the Alteration of the Gut Microbiota Caused by D-Galactosamine

**Figure 3:**
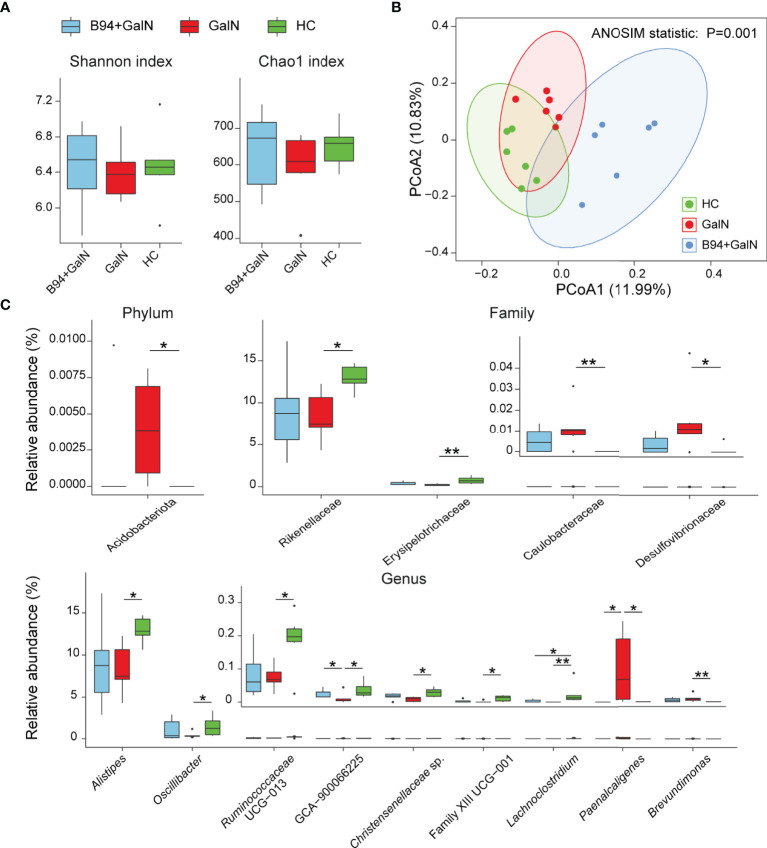
B94 treatment alleviates D-galactosamine-induced dysbiosis of the gut microbiota. **(A)** Box plot of species richness and flora diversity estimated based on the Shannon indexes and Chao1 indexes. **(B)** Two-dimensional PCoA plot based on the unweighted UniFrac matrix confirmed by ANOSIM. **(C)** Alterations in the relative abundances of bacterial taxa in the GalN, B94+GalN, and HC groups at the phylum, family and genus levels. * *P* < 0.05; ** *P* < 0.01.

A total of 888,670 reads from 18 faecal samples were obtained by 16S rRNA sequencing. For alpha diversity, the community biodiversity and community richness, as indicated by the Shannon index and Chao1 index, were not significantly different among the HC, GalN and B94+GalN groups (**Figure 3A**). For beta diversity, the principle coordinate analysis (PCoA) showed that the microbiota profiles of the three groups could be clearly separated (**Figure 3B**), and the results of analysis of similarities (ANOSIM) confirmed that there was a significant intergroup difference in the bacterial composition among the three groups (*P* = 0.001).

We further performed a statistical analysis of the changes in the relative abundances of individual bacterial taxa among the HC, GalN and B94+GalN groups. Compared with those in the HC group, the phylum Acidobacteriota, the families *Caulobacteraceae* and *Desulfovibrionaceae* and the genus *Brevundimonas*, which belong to the phylum Pseudomonadota, were enriched in the GalN group. Conversely, the family *Erysipelotrichaceae* and the genera *Lachnoclostridium*, *Christensenellaceae* sp., uncultured *Ruminococcus* subsp. (GCA 900066225), *Oscillibacter*, *Ruminococcaceae* UCG-013 and Clostridiales Family XIII sp., which belong to the phylum Bacillota, and the family *Rikenellaceae* and genus *Alistipes*, which belong to the phylum Bacteroidota, were depleted in the GalN group (**Figure 3C**).

B94 treatment partially ameliorated the changes in the gut microbiota induced by D-galactosamine. First, compared to that in the GalN group, the genus *Paenalcaligenes*, which belongs to the phylum Pseudomonadota, was depleted in the B94+GalN group, and the genus GCA-900066225, which belongs to the phylum Bacillota, was enriched (**Figure 3C**). Second, B94 mitigated the changes in the relative abundances of the following taxa caused by D-galactosamine so that the differences with the HC group were no longer significant, including the phylum Acidobacteriota, the families *Caulobacteraceae* and *Desulfovibrionaceae* and genus *Brevundimonas*, which belong to phylum Pseudomonadota, the family *Erysipelotrichaceae* and genera *Christensenellaceae* sp., *Oscillibacter*, *Ruminococcaceae* UCG-013 and Clostridiales Family XIII sp., which belong to the phylum Bacillota, and the family *Rikenellaceae* and genus *Alistipes*, which belong to the phylum Bacteroidota (**Figure 3C**).

**Figure 4:**
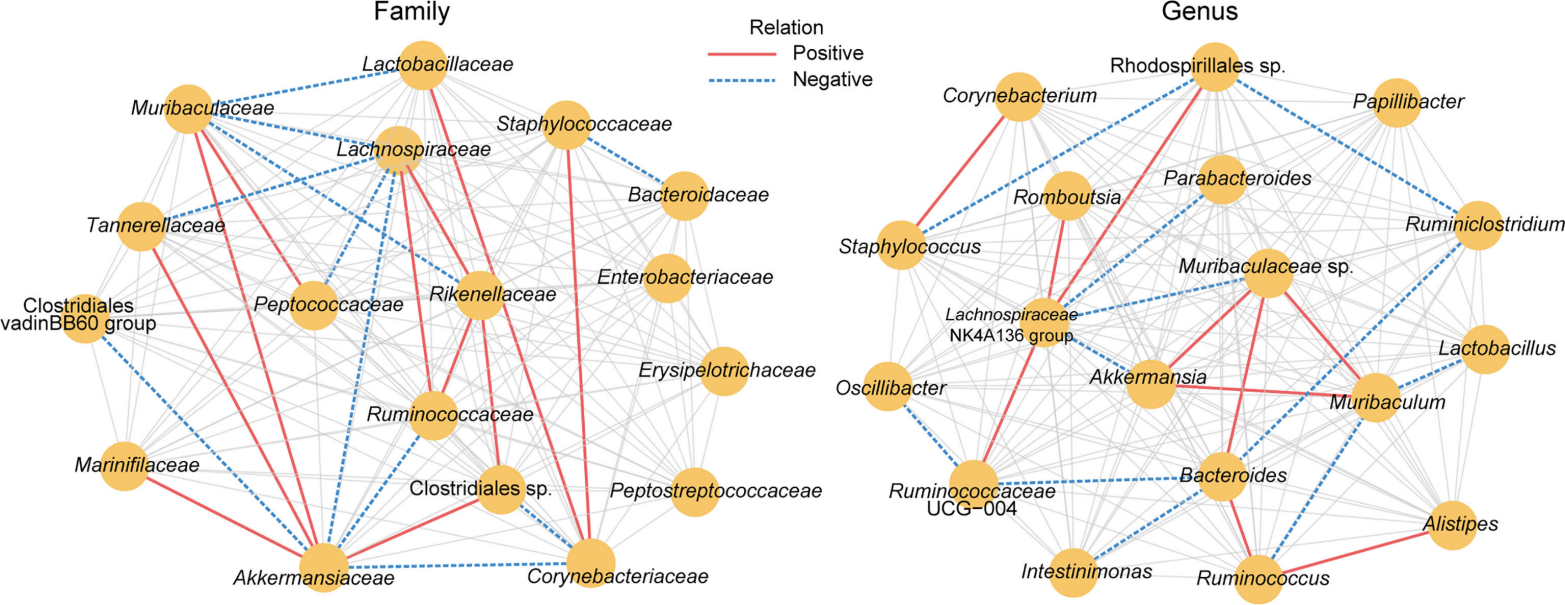
The microbial association network influenced by B94 treatment was inferred by using the SparCC method. Significant correlations were screened (|R| >0.2 and *P* < 0.05) and are displayed with solid red lines (positive) and dashed blue lines (negative).

To study the microbial association network, the correlation of OTUs at the family and genus levels from the three groups was analysed using the Sparse Correlations for Compositional data (SparCC) method. The absolute value of the correlation coefficient R >0.2 and *P* < 0.05 were used as screening thresholds. As a result, bacterial taxa belonging to the phylum Bacteroidota and Bacillota, such as *Muribaculaceae*, *Ruminococcaceae*, *Rikenellaceae*, *Lachnospiraceae*, *Lactobacillaceae*, *Bacteroidaceae*, *Peptococcaceae*, and *Staphylococcaceae*, are located at the core of the network (**Figure 4**), suggesting their important role in the gut microbiota changes related to B94 treatment. Additionally, B94 treatment-ameliorated microbial taxa, such as the family *Rikenellaceae* and the genus *Alistipes*, were positively correlated with *Ruminococcaceae* (or *Ruminococcus*), Clostridiales sp. and *Lachnospiraceae*. This is in line with our finding that B94 mitigated the D-galactosamine-induced changes in the abundances of taxa belonging to the phyla Bacteroidota and Bacillota.

### *Bifidobacterium animalis* B94 Improved Gut Metabolic Disorders

**Figure 5:**
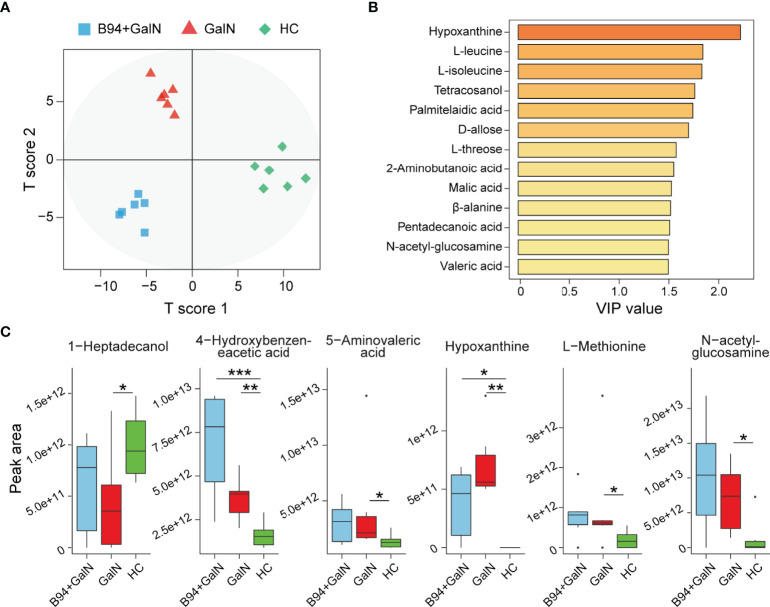
B94 treatment alleviates D-galactosamine-induced gut metabolism disorder. **(A)** OPLS-DA plot illustrating clear separation of the gut metabolic profiles of the GalN, B94+GalN, and HC groups. **(B)** VIP values of 13 metabolites with the highest contribution to the separation of the three groups in the OPLS-DA model. **(C)** Levels of six differentially distributed metabolites in the three groups. * *P* < 0.05; ** *P* < 0.01; *** *P* < 0.001.

We investigated the effect of B94 on D-galactosamine-induced gut metabolic disorder based on gas chromatography-mass spectrometry (GC-MS) analysis. In total, 105 metabolites were identified from the three groups. The results of orthogonal projections to latent structures discriminant analysis (OPLS-DA) showed that the HC, GalN and B94+GalN groups were clearly separated (**Figure 5A**), indicating that their metabolome profiles were significantly different. The variable importance for projection (VIP) values of 13 metabolites (hypoxanthine, L-leucine, L-isoleucine tetracosanol, palmitelaidic acid, D-allose, L-threose, 2-aminobutanoic acid, malic acid, β-alanine, pentadecanoic acid, N-acetyl glucosamine and valeric acid) were greater than 1.5, suggesting their contributions to differentiating the metabolome profiles of the three groups in this OPLS-DA model (**Figure 5B**).

Next, we compared the levels of each faecal metabolite among the different groups. Compared to that in the HC group, 1-heptadecanol was significantly depleted in the GalN group, whereas 4-hydroxybenzene acetic acid, 5-aminovaleric acid, hypoxanthine, L-methionine and N-acetyl glucosamine were enriched. After B94 treatment, the D-galactosamine-induced changes in the levels of 1-heptadecanol, 5-aminovaleric acid, L-methionine and N-acetyl glucosamine were no longer significant (**Figure 5C**).

### The Beneficial Effects of B94 Pretreatment on the Gut Microbiota, Metabolism, Serum Liver Function Indicators and Cytokines Were Closely Correlated

**Figure 6:**
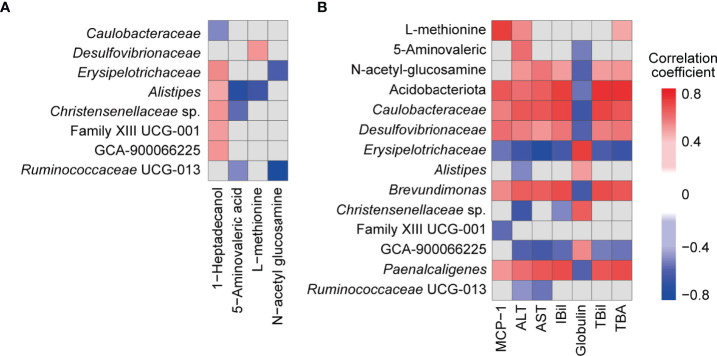
Associations among faecal bacteria, faecal metabolites, and blood indicators influenced by B94 treatment (|R| >0.4 and *P* < 0.05). **(A)** Correlation of B94-influenced faecal bacteria with faecal metabolites. **(B)** Correlation of B94-influenced faecal bacteria and metabolites with liver function indicators and cytokines.

To explore the potential relationship between the gut microbiota and metabolism, we performed an association analysis using Spearman’s rank correlation. Both the absolute value of the correlation coefficient R > 0.4 and *P* < 0.05 were used as the screening threshold to identify significant results. Among the metabolites, 1-heptadecanol was widely associated with microbes, such as positively correlated with *Alistipes*, *Christensenellaceae* sp., Clostridiales Family XIII sp., GCA-900066225, and *Erysipelotrichaceae*, and negatively correlated with *Caulobacteraceae* (**Figure 6A**). Moreover, *Alistipes* was negatively correlated with 5-aminovaleric acid (R = -0.72, *P* = 8.49E-04) and L-methionine (R = -0.63, *P* = 4.99E-03); *Erysipelotrichaceae* (R = -0.60, *P* = 8.98E-03) and *Ruminococcaceae* UCG-013 (R = -0.83, *P* = 1.78E-05) were negatively correlated with N-acetyl glucosamine; L-methionine was positively correlated with *Desulfovibrionaceae*; 5-aminovaleric acid was negatively correlated with *Christensenellaceae* sp. and *Ruminococcaceae* UCG-013 (**Figure 6A**).

Next, we analysed the association of gut microbes and metabolites with liver function indicators and cytokines. First, the TBil, IBil, ALT, AST and TBA levels were positively correlated with the metabolite N-acetyl glucosamine and microbes *Caulobacteraceae*, *Desulfovibrionaceae*, *Brevundimonas* and *Paenalcaligenes*, which belong to the phylum Acidobacteriota or Pseudomonadota, and were negatively correlated with *Erysipelotrichaceae* and GCA-900066225, which belong to the phylum Bacillota (**Figure 6B**). However, globulin was correlated with these microbes and metabolites in the opposite way as TBA and the others mentioned above. Additionally, globulin was negatively correlated with 5-aminovaleric acid and positively correlated with *Alistipes* and *Christensenellaceae* sp.; ALT was negatively correlated with *Alistipes*, *Christensenellaceae* sp., and *Ruminococcaceae* UCG-013 but positively correlated with 5-aminovaleric acid and L-methionine; AST was positively correlated with *Ruminococcaceae* UCG-013; IBil was negatively correlated with *Christensenellaceae* sp.; L-methionine was positively correlated with TBA. Second, the serum cytokine MCP-1 was positively correlated with Acidobacteriota, *Caulobacteraceae*, *Desulfovibrionaceae*, *Brevundimonas*, *Paenalcaligenes* and L-methionine but negatively correlated with *Erysipelotrichaceae* and Clostridiales Family XIII sp. (**Figure 6B**).

## Discussion

Liver failure refers to serious liver injury caused by various factors that is difficult to treat and has a poor prognosis. Liver injury is closely related to the gut microbiota. Alterations in gut microbiota have an important influence on the occurrence and development of liver failure. Probiotics have an important role in regulating the gut microbiota to prevent diseases; however, the role and mechanism of most probiotics in the prevention and treatment of liver failure are not clear. In this study, we found that B94 alleviated D-galactosamine-induced abnormal liver function, elevated MCP-1 levels in serum, and attenuated pathological damage to the colon and liver. B94 also ameliorated D-galactosamine-induced alterations in the gut microbiota and metabolome. These results provide a basis for the use of B94 in the prevention and treatment of liver injury.

Our results suggest that B94 extensively ameliorated D-galactosamine-induced liver function indicator abnormalities. The liver is the main site of globulin synthesis, and damage to hepatocytes decreases globulin levels. ALT and AST are important liver disease indicators that are mainly distributed in hepatocytes and will rapidly enter the bloodstream when hepatocytes are damaged. Total bile acids are a group of cholesterol metabolites in hepatic catabolism and intestinal-hepatic circulation, and bilirubin is the main metabolite of iron porphyrin compounds in the body. The serum TBA and bilirubin concentrations are elevated when liver disease or obstruction occurs, which can sensitively reflect liver function. B94 administration can significantly ameliorate the elevation in albumin/globulin ratio, ALT, AST, TBA, TBil and IBil, the decrease in globulin levels, and reduce liver and intestinal damage caused by D-galactosamine. This finding shows that B94 has a good ability to prevent liver injury.

Our results suggest that B94 ameliorated D-galactosamine-induced gut microbiota dysbiosis. First, B94 slowed the depletion of potentially beneficial gut bacteria. For example, the beneficial effects of *Ruminococcaceae* UCG-013 and uncultured *Ruminococcus* subsp. (GCA-900066225) on health include degradation of resistant starch and stabilization of the intestinal barrier (Cann et al., 2016). The relative abundance of *Christensenellaceae* in the human gut is inversely related to host body mass index (BMI), obesity and inflammatory bowel disease (Waters and Ley, 2019). *Odoribacteraceae* has been found in a group of Japanese people who have lived over a century and can produce isoallo-lithocholic acid, which is one of the most potent antimicrobial agents selectively against gram-positive microbes such as *Clostridium difficile* (Jiang et al., 2021). Our correlation analysis results are consistent with this finding; for example, *Erysipelotrichaceae* and GCA-900066225 were negatively correlated with serum TBil, IBil, ALT and TBA; *Alistipes*, *Christensenellaceae* sp. and *Ruminococcaceae* UCG-013 were negatively correlated with ALT; *Erysipelotrichaceae* and Family XIII UCG-001 were negatively correlated with MCP-1. Second, B94 slowed the increase in conditionally pathogenic bacteria. For example, *Paenalcaligenes hominis*, particularly its extracellular vesicles, is a risk factor for vagus nerve-mediated cognitive impairment (Lee et al., 2020); members of *Caulobacteraceae*, such as *Brevundimonas*, play a role in infections (Liu et al., 2021); and *Desulfovibrionaceae* are sulfate-reducing and endotoxin-producing bacteria (Liechty et al., 2020). In line with this finding, we found that serum TBil, IBil, TBA, ALT, AST and the cytokine MCP-1 were positively correlated with *Caulobacteraceae*, *Desulfovibrionaceae*, *Brevundimonas*, and *Paenalcaligenes* in the gut. Moreover, the role of some of the B94-regulated gut microbes in health and disease needs to be further explored. For example, Acidobacteriota inhabit a wide variety of terrestrial and aquatic habitats and are particularly abundant in acidic soils, and Acidobacteriota has been reported to be depleted in patients with primary biliary cirrhosis (Lv et al., 2016). In this study, the B94-regulated taxon *Alistipes*, which belongs to the phylum Bacteroidota, has been reported to correlate not only with protection against diseases such as colitis, autism spectrum disorder, and various liver and cardiovascular fibrotic disorders but also with the development of diseases such as anxiety, myalgic encephalomyelitis/chronic fatigue syndrome, depression, pervasive developmental disorder-not otherwise specified (PDD-NOS) and colorectal cancer (CRC) (Parker et al., 2020). In summary, regulation of the gut microbiota is one of the important means by which B94 performs its function in the prevention and treatment of liver injury.

Stool contains products of cometabolism between the gut microbiota and the host, which act as a bridge for the interaction between the host and the microbes. We found that B94 pretreatment reduced the excretion of potentially beneficial metabolites in the faeces caused by D-galactosamine. For example, 5-aminovaleric acid is an analogue of γ-aminobutyric acid (GABA), which has been reported to inhibit GABA uptake and GABA aminotransferase activity (Lv et al., 2021c). L-methionine, an essential amino acid, is not only involved in protein synthesis but is also the main source of methyl groups in methyl transfer reactions and has important physiological functions, such as inhibition of fat accumulation and enhancement of the immune response (Shim et al., 2017). N-acetyl glucosamine is the basic unit of many important polysaccharides in biological cells and plays important roles in wound healing, antioxidant and immune-modulating effects, and regulation of liver function (Jiang et al., 2018). In addition, B94 pretreatment alleviated the reduction in the abundance of 1-heptadecanol in faeces caused by D-galactosamine. 1-heptadecanol is a long-chain primary alcohol with antimicrobial and anti-inflammatory potential observed in plants such as *Solena amplexicaulis* leaves (Lv et al., 2021b). In conclusion, B94 pretreatment slowed the disturbance of gut metabolism caused by D-galactosamine, which in turn was beneficial in reducing liver and intestinal damage.

Although there are many *Lactobacillus* and *Bifidobacterium* species, which are the major probiotics worldwide, only a small number of strains have been reported to have a role in preventing liver injury or failure. For example, in addition to the strain B94 that we found, *Lactobacillus salivarius* LI01, *Bifidobacterium adolescentis* CGMCC 15058, *Bifidobacterium longum* R0175, *Bifidobacterium pseudocatenulatum* LI09, *Bifidobacterium catenulatum* LI10, *Lactobacillus helveticus* R0052, *Lactobacillus reuteri* DSM 17938, *Lactobacillus acidophilus* LA14, and *Lactobacillus casei* Shirota have also been reported to have effects against liver injury (Osman et al., 2007; Wang et al., 2013; Lv et al., 2014; Fang et al., 2017; Li et al., 2019; Wang et al., 2019; Wang et al., 2020; Lv et al., 2021a; Jiang et al., 2021; Yan et al., 2021). There are certain commonalities in the effects of each strain on liver function, immunity and metabolism; for example, B94, LI01, DSM 17938, LA14, LI09 and LI10 lowered ALT and AST levels (Lv et al., 2014; Fang et al., 2017; Lv et al., 2021a; Jiang et al., 2021); B94, CGMCC 15058, R0175, Shirota; Li09, LI10, and LA14 lowered TBA levels (Fang et al., 2017; Li et al., 2019; Wang et al., 2020; Lv et al., 2021a; Yan et al., 2021); LI01 and LA14 lowered ALP levels (Lv et al., 2014; Lv et al., 2021a); and B94 and R0175 lowered TBil levels (Wang et al., 2020). Although these results regarding the prevention and treatment of liver injury are based on animal models and need further clinical validation, it is promising that most of these strains are commonly used probiotics, which have a good safety profile and are easily available to patients, thus providing a significant contribution to the prevention and treatment of liver injury.

In conclusion, this research examined the effect of *Bifidobacterium animalis* B94 on D-galactosamine-induced liver injury in rats. *Bifidobacterium animalis* B94 significantly reduced the elevated levels of ALT and AST induced by D-galactosamine, improved the gut microbiota as well as metabolic dysbiosis, reduced pathological abnormalities in liver and intestinal tissues, and has important potential applications for liver injury and liver failure.

## Data Availability Statement

The datasets presented in this study can be found in online repositories. The names of the repository/repositories and accession number(s) can be found in the article/**Supplementary Material**.

## Ethics Statement

The animal study was reviewed and approved by Animal Experimentation Ethics Committee of Zhejiang University.

## Author Contributions

TZ and LN conceived and designed the study. TZ, LN, JW, ZY, YZ, and SW performed the experiments and analysed the data. TZ, LN, and ZC wrote the manuscript. All authors contributed to the article and approved the submitted version.

## Funding

This work is supported by the National Key Research and Development Program of China (No. 2021YFA1301102).

## Conflict of Interest

The authors declare that the research was conducted in the absence of any commercial or financial relationships that could be construed as a potential conflict of interest.

## Publisher’s Note

All claims expressed in this article are solely those of the authors and do not necessarily represent those of their affiliated organizations, or those of the publisher, the editors and the reviewers. Any product that may be evaluated in this article, or claim that may be made by its manufacturer, is not guaranteed or endorsed by the publisher.

## Abbreviations

HC, Healthy control; GalN, Galactosamine; TBA, Total bile acid; TBil, Total bilirubin; DBil, Direct bilirubin; IBil, Indirect bilirubin; ALT, Alanine transaminase; AST, Aspartate transaminase; ALP, Alkaline phosphatase; GGT, γ-glutamyl transferase; GPDA, Glycylproline dipeptidyl aminopeptidase; MIP-1α, Macrophage inflammatory protein-1α; MCP-1, Macrophage chemoattractant protein-1.
