# Supplementary figures and images for: Effect and Mechanism of Bifidobacterium animalis B94 in the Prevention and Treatment of Liver Injury in Rats
Source: Front Cell Infect Microbiol. 2022 Jun 29;12:914684. doi: 10.3389/fcimb.2022.914684 (PMC9277360; doi:10.3389/fcimb.2022.914684)

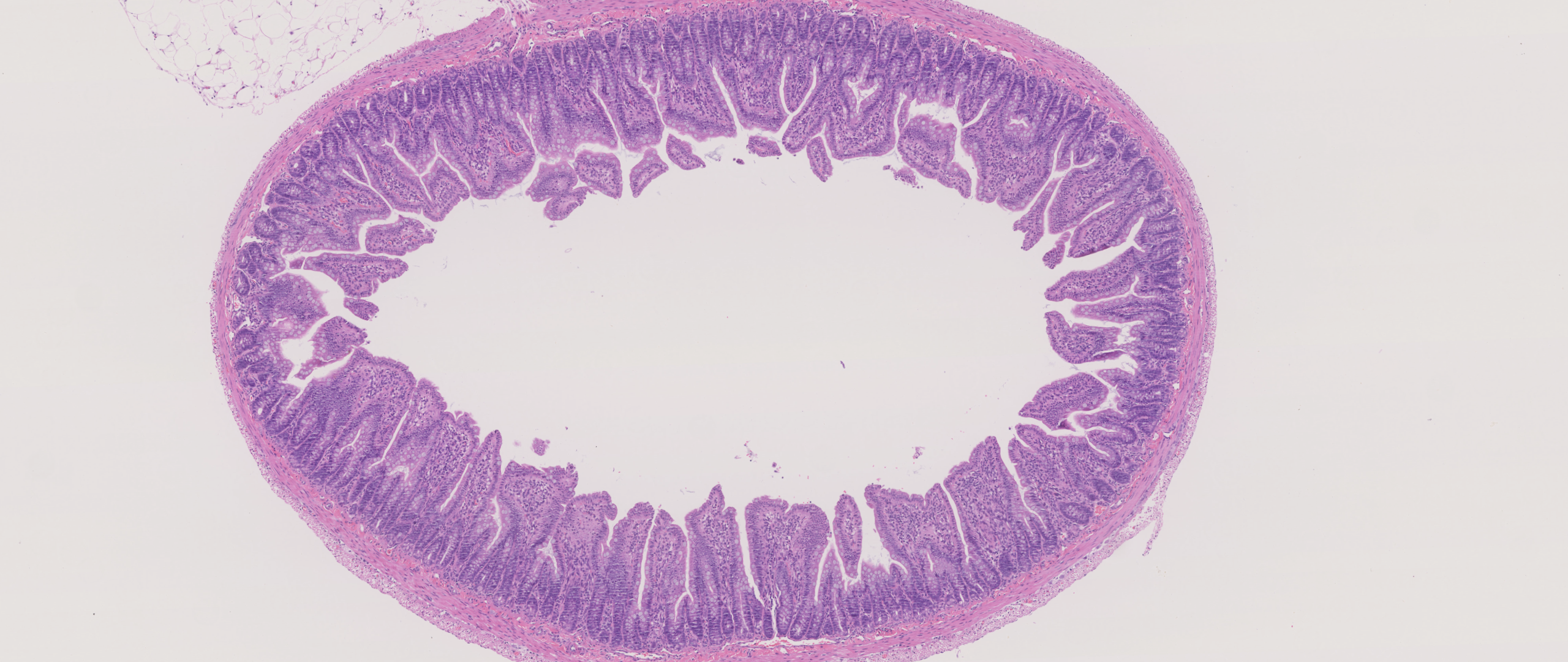

Supplement: Supplementary file 1 [file Image_1.tif]

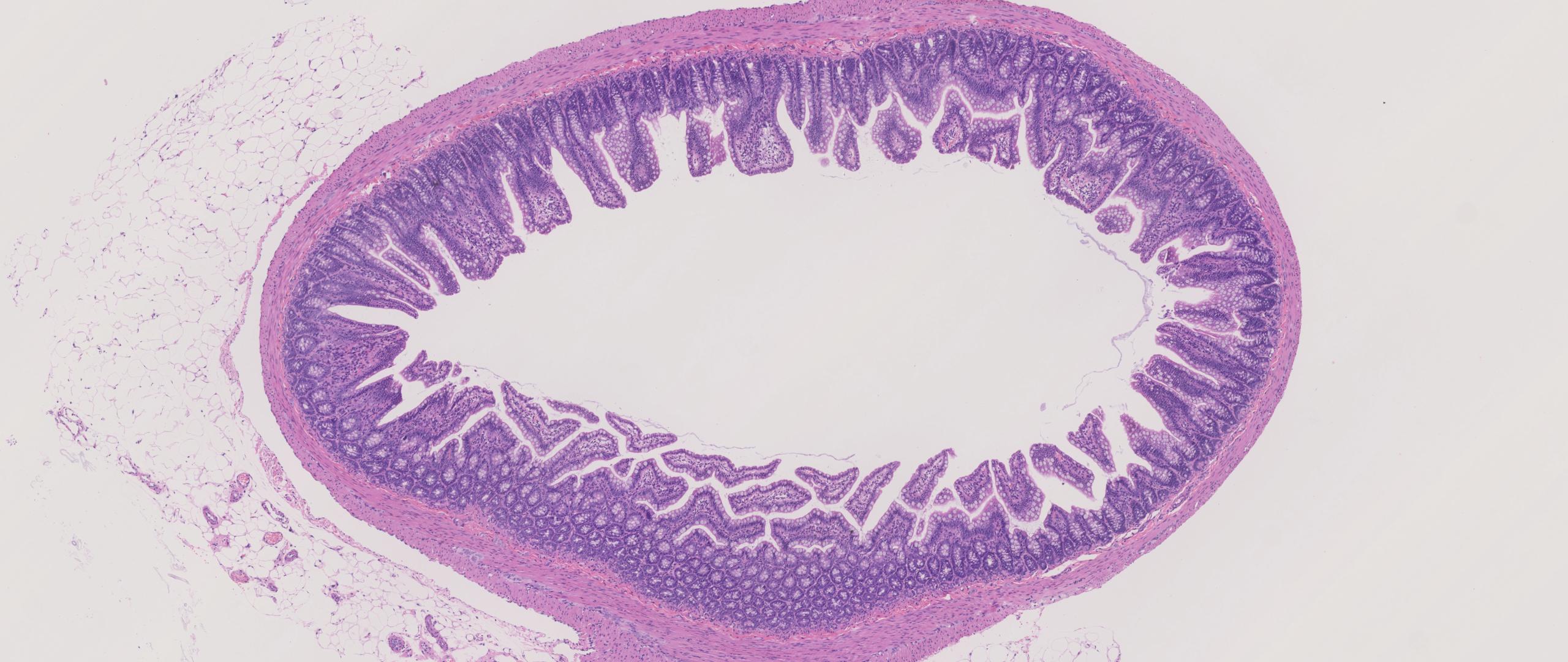

Supplement: Supplementary file 2 [file Image_2.tif]

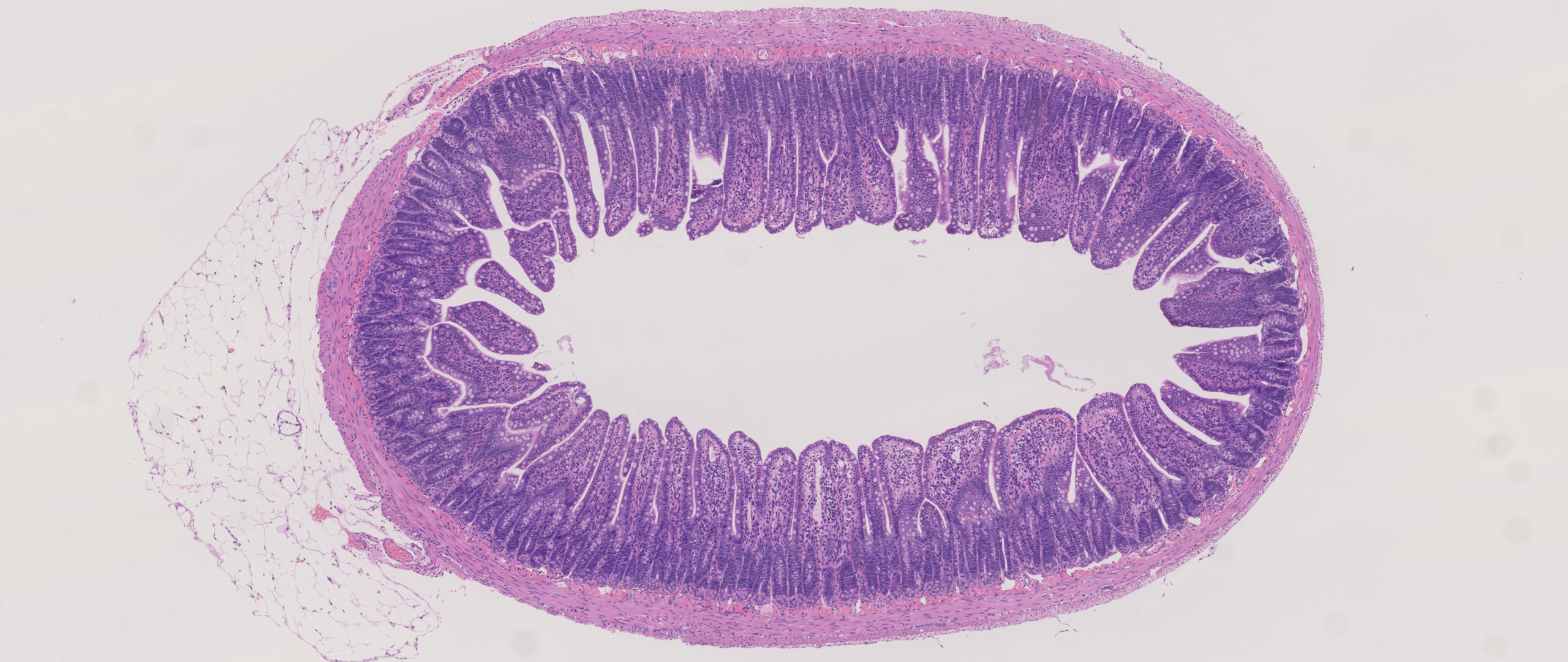

Supplement: Supplementary file 3 [file Image_3.tif]

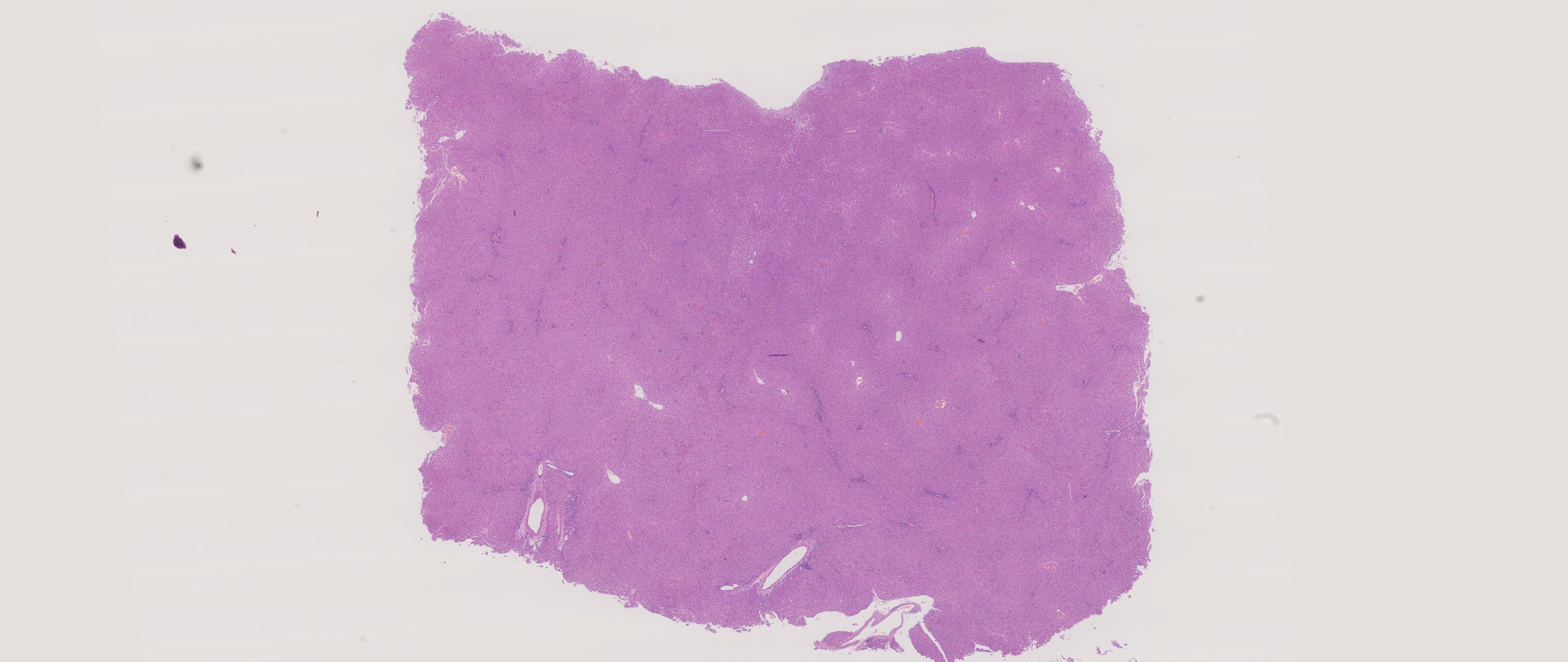

Supplement: Supplementary file 4 [file Image_4.tif]

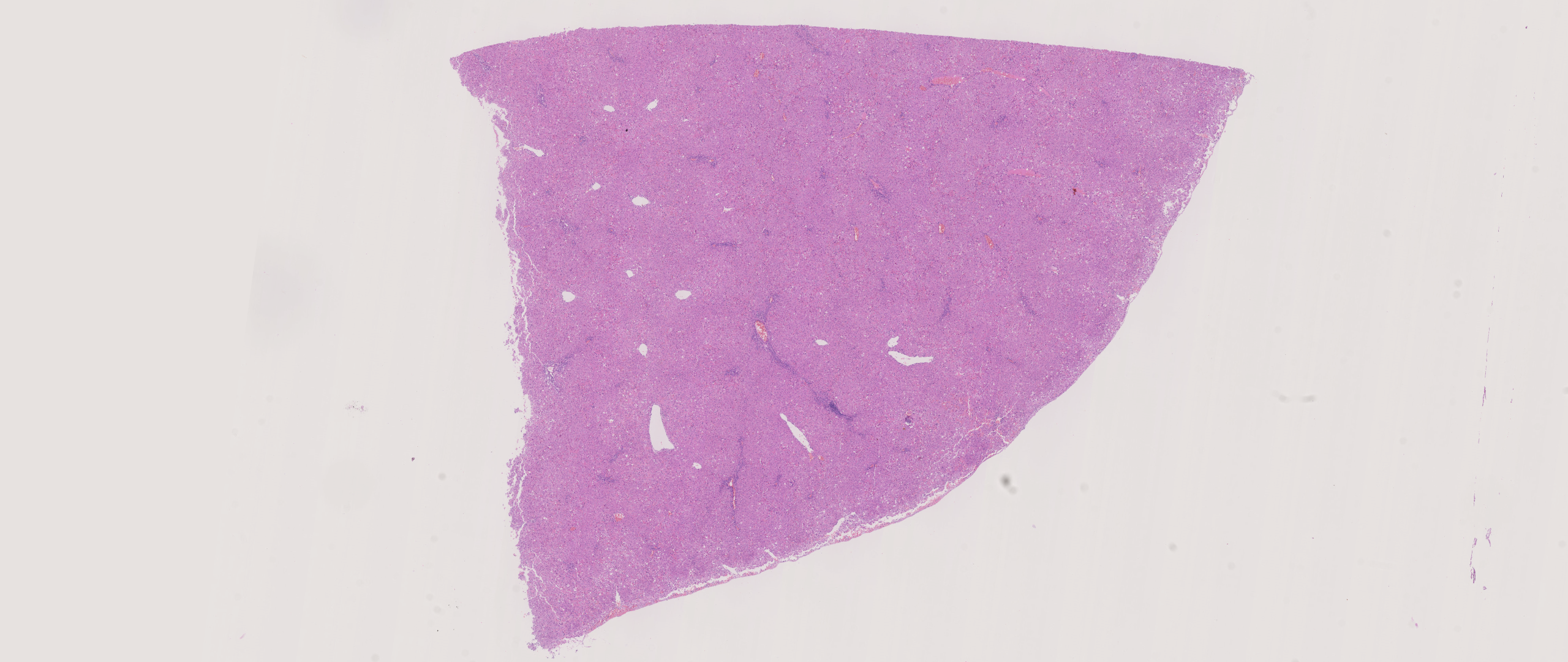

Supplement: Supplementary file 5 [file Image_5.tif]

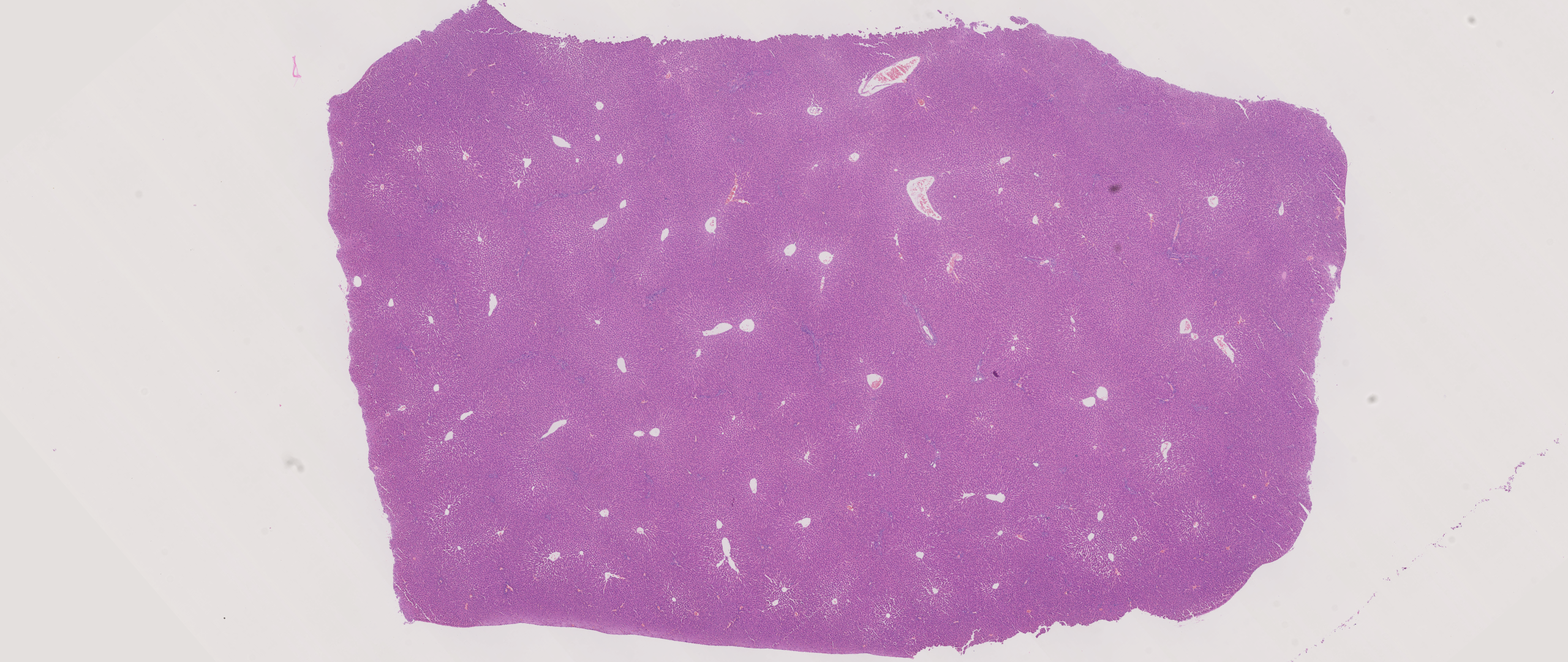

Supplement: Supplementary file 6 [file Image_6.tif]
